# ComB proteins expression levels determine *Helicobacter pylori* competence capacity

**DOI:** 10.1038/srep41495

**Published:** 2017-01-27

**Authors:** Christopher Corbinais, Aurélie Mathieu, Prashant P. Damke, Thierry Kortulewski, Didier Busso, Mariano Prado-Acosta, J. Pablo Radicella, Stéphanie Marsin

**Affiliations:** 1 Institute of Molecular and Cellular Radiobiology, CEA, F-92265 Fontenay aux Roses, France; 2 INSERM, U967, F-92265 Fontenay-aux-Roses, France; 3 Universités Paris Diderot et Paris Sud, UMR967, F-92265 Fontenay-aux-Roses, France

## Abstract

*Helicobacter pylori* chronically colonises half of the world’s human population and is the main cause of ulcers and gastric cancers. Its prevalence and the increase in antibiotic resistance observed recently reflect the high genetic adaptability of this pathogen. Together with high mutation rates and an efficient DNA recombination system, horizontal gene transfer through natural competence makes of *H. pylori* one of the most genetically diverse bacteria. We show here that transformation capacity is enhanced in strains defective for *recN,* extending previous work with other homologous recombination genes. However, inactivation of either *mutY* or *polA* has no effect on DNA transformation, suggesting that natural competence can be boosted in *H. pylori* by the persistence of DNA breaks but not by enhanced mutagenesis. The transformation efficiency of the different DNA repair impaired strains correlates with the number of transforming DNA foci formed on the cell surface and with the expression of *comB8* and *comB10* competence genes. Overexpression of the *comB6-B10* operon is sufficient to increase the transformation capacity of a wild type strain, indicating that the ComB complex, present in the bacterial wall and essential for DNA uptake, can be a limiting factor for transformation efficiency.

*Helicobacter pylori* chronically colonises the stomach of approximately half of the world’s population. The infection triggers chronic inflammation, remaining in most cases asymptomatic but that can evolve into serious pathologies such as peptic ulcer or gastric cancer^1^,^2^. *H. pylori* infection can be eradicated by antibiotics treatment, but the last decade has seen dramatic increases in the appearance of resistant strains^3^. The amazing genome plasticity observed in comparative analysis of isolates could explain the high adaptability of *H. pylori* and its success as a pathogen^4^,^5^. Several reasons can be pointed out to explain the high genetic variability of *H. pylori*. This species presents one of the highest mutation frequencies known^6^ combined with abundant chromosomal rearrangements^7^. The widespread presence of strain-specific genes suggests that horizontal gene transfer (HGT) plays a prominent role in the genetic diversity of the species. First evidence for natural transformation resulting in HGT in *H. pylori* was reported in 1990^8^ and further confirmed to occur *in vivo* during human infection^5^,^9^,^10^. The transformation capacity reported for different *H. pylori* isolates varies enormously. Transformation frequencies of up to 50% have been described under laboratory conditions^11^,^12^. This amazing transformation efficiency is likely to contribute to the increase in antibiotic resistance spreading observed the last ten years^3^,^13^.

Natural transformation has been studied for many years in several bacterial models, originally in the Gram-positive *Streptococcus pneumoniae* and *Bacillus subtilis* (for a review see ref. 14). In all the systems studied so far, including Gram-negative species, transforming double-stranded DNA (dsDNA) from the environment is imported through the bacterial membrane and processed to yield single-stranded DNA (ssDNA) before its internalisation into the cytoplasm. There, ssDNA is recognised by the Homologous Recombination (HR) machinery and integrated into the host chromosome. The loading of the RecA recombinase is facilitated by DprA, a recombination mediator protein dedicated to DNA transformation^15^. In the majority of bacteria, Gram-positive as well as Gram-negative, internalisation occurs through protein complexes homologue to Type 4 Pilus (T4P)^14^. However, in *H. pylori* this T4P is replaced by conjugation-type proteins with homology to Type 4 secretion system components from *Agrobacterium tumefaciens.* Computational and genetic analysis revealed that the membrane complex is encoded in *H. pylori* by two operons, *comB2-B4* and *comB6-B10,* named after the *A. tumefaciens* orthologues^16^,^17^. This complex is essential for the entrance of transforming DNA (tDNA) into the periplasm. Based on the VirB/D system, the ComB7, ComB9 and ComB10 proteins are proposed to be involved in the formation of the outer membrane channel, while ComB6 and ComB8 are supposed to be anchored to the inner membrane^18^. The passage through the inner membrane is then carried out by the ComEC protein^11^,^12^,^19^. It has been proposed that DNA transport between the cell surface and the cytoplam in *H. pylori* occurs in two distinct steps^12^.

The conditions inducing natural competence for transformation, the fraction of the population affected and the growth phase in which this occurs vary from species to species. In most cases, natural competence for transformation is induced through complex regulatory networks responding to specific environmental cues (for reviews see refs 14, 20 and 21). *H. pylori,* on the other hand, was first presented as being constitutively competent^8^. Consistently, no orthologues for the competence regulatory genes studied in the other models were identified in its genome. However, it was shown that competence varies during growth in *H. pylori*^22^ and that modification of O_2_ and CO_2_ conditions can be determinant for DNA transformation efficiency in *H. pylori*^23^. More recent studies have shown that pH and oxidative stress are critical for defining *H. pylori* DNA uptake capacity. Indeed, DNA uptake is reversibly shut down below pH 6.5^24^. In agreement with the reported increase in transformation capacity of strains deficient in the HR repair genes *addA, addB* and *recO*^25^,^26^, accumulation of DNA damage was proposed to boost transformation^27^. Interestingly, DNA damage caused by UV, fluoroquinolones or mitomycin C was shown to induce competence in the Gram-positive *S. pneumoniae*^28^,^29^ and in the Gram-negative *Legionella pneumophila*^30^. In the case of *S. pneumoniae*, the mutational burden in coding sequences caused by inactivation of a DNA repair pathway induces competence^31^. Because translational errors were also shown to induce competence^32^, the effect of mutations in coding sequences on competence was proposed to be a response to the presence of misfolded proteins.

Here, we used a genetic approach to explore the impact of DNA damage on *H. pylori* competence. We show that transformation capacity is induced in strains mutated in HR genes such as *addA, recO* or *recN*, but not in the *polA* and *mutY* genes involved in other DNA repair pathways. Furthermore, transformation efficiency correlates with DNA uptake capacity reflected in the formation of tDNA foci on the bacteria and, ultimately, with the *comB6-B10* operon expression levels.

## Results

### DNA damage, mutations and bacterial transformation capacity

**Figure 1:**
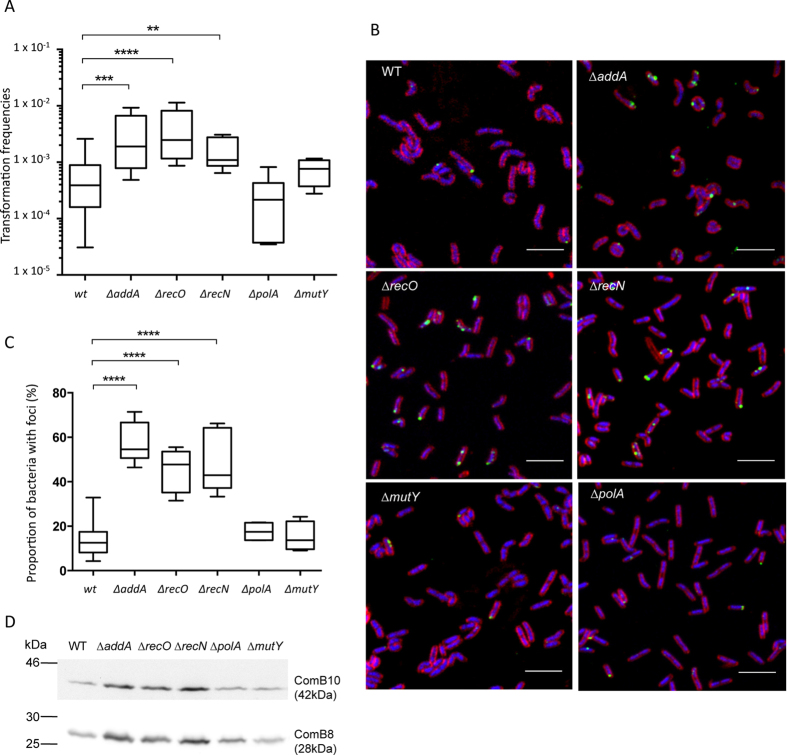
Transformation frequencies, foci formation and ComB expression in DNA repair mutants. (**A**) Transformation frequencies. The bar represents the median, the boxes display the inner quartile range and the whiskers represent the minimum and maximum values. Number of experiments and p value determined with Mann-Whitney U test are indicated in Table 1. (**B**) Microscopy images of fixed bacteria. For each of the indicated strains, merged images are presented with red for FMX64, blue for DAPI and green for ATTO488. Scale bars correspond to 5 μm. (**C**) Proportion of bacteria with foci. At least 3 independent experiments were analyzed and more than 1200 bacteria were counted for each strain. The percentage of bacteria harboring foci are indicated for the different strains. The bar represents the median, the boxes display the inner quartile range and the whiskers represent the minimum and maximum values. (**D**) Western blot of wild-type and different mutants against ComB10 (upper line) and ComB8 (lower line).

Previous reports showed that strains deficient in either RecOR or AddAB recombination mediator complexes display increased transformation frequencies^25^,^26^. Interestingly, the expression of competence genes in *H. pylori,* among which several *comB,* was found to be elevated in *addA* mutants as well as after treatment with ciprofloxacin, an antibiotic that inhibits several DNA processing enzymes and therefore generates DNA breaks^27^. These results suggested that the persistence of DNA strand breaks in the bacterial chromosome triggers an increase in transformation capacity. We confirmed the enhanced transformation frequencies of *recO* and *addA* mutants (Table 1 and Fig. 1A), albeit with different values likely due to improved bacterial culture conditions and transformation protocol with which we obtain a 10-fold increase in the yield of recombinants for a *wt* strain (see Methods). Because *addA* strains are slow growers and to avoid a possible bias in transformation efficiencies due to the heterogeneity of phase growth status characteristic of plate cultures, we repeated the experiment for *wt* and *addA* strains in the same growth phase using liquid cultures in controlled conditions. In that case we obtained a 6-fold increase in recombination frequencies for the mutant strain compared to the *wt* (data not shown). Consistently with the hypothesis that persistence of strand breaks can induce transformation, disruption of *recN*, another gene involved in strand break repair^33^, also resulted in a significant increase of recombinant frequencies when bacteria were transformed with genomic DNA from a streptomycin resistant strain (Table 1 and Fig. 1A). To test whether the mutation burden also affected transformation capacity, as proposed for *S. pneumoniae*^31^, we used as recipients strains defective in DNA repair pathways affecting mutation frequencies. We have previously shown that, due to its role in translesion synthesis, a deficiency in DNA Polymerase I leads to a 10-fold decrease in spontaneous mutation rates^34^. Despite this, the transformation capacity of a *polA* mutant strain was not significantly different from that of the *wt* (Fig. 1A and Table 1). We then tested a strain defective in MutY, a base excision repair protein involved in the avoidance of mutations induced by 8-oxoguanine^35^. Inactivation of *mutY,* while increasing mutation frequencies by 10-fold^35^,^36^,^37^, did not change significantly the transformation efficiency compared to that of the *wt* strain (Table 1 and Fig. 1A). Taken together, these results suggest that in *H. pylori*, DNA breaks, but not mutations, induce transformation capacity.

### Transformation capacity, tDNA foci formation and ComB expression level in DNA repair mutants

We next analysed whether the variations in transformation frequencies determined by the disruption of DNA repair genes were due to changes in the capacity of the bacteria to capture exogenous DNA or to process it once inside the cell. For that purpose, we used transforming DNA (tDNA) labelled with fluorescent ATTO-dUTP. ATTO-labelled tDNA forms ComB-dependent foci within the periplasm. The DNA present in the foci is eventually internalised into the cytoplasm and gives rise to recombinant bacteria^11^. When we analysed tDNA foci formation in the DNA strand break repair deficient strains, we observed in all cases an increase in the frequency of bacteria displaying foci (Fig. 1B and C, Table 2). These results indicate a correlation between the fraction of cells displaying tDNA foci, therefore DNA uptake, and the transformation capacity of the strain. It is worth noting that while this manuscript was in preparation a similar correlation between these two parameters was described by Kruger *et al*.^24^. Because the formation of tDNA foci requires the expression of a functional ComB complex (Tables 1 and 2 and refs 11,12) and *comB* genes RNA levels were found to be up-regulated in *addA* mutants^27^, we analysed the relative expression levels of ComB8 and ComB10 proteins in the different DNA repair mutant strains. As shown in Fig. 1D, the enhanced foci formation and transformation capacity of the recombination-deficient strains were associated with an increase in the expression of ComB8 and ComB10. In the case of *mutY* and *polA* strains, in which we did not observe variations in transformation frequencies nor in foci formation compared to the wild type (Table 2 and Fig. 1A and C), the levels of ComB8 and ComB10 were not significantly altered. In all the mutants tested here, the transformation efficiencies were positively correlated with the frequencies of bacteria with foci (r^2^ = 0.80; p = 0.0065) (Fig. S1B) and with the ComB8 expression levels (r^2^ = 0.65; p = 0.0299) (Fig. S1C).

### Overexpression of *comB6-B10* operon is sufficient to increase transformation

**Figure 2:**
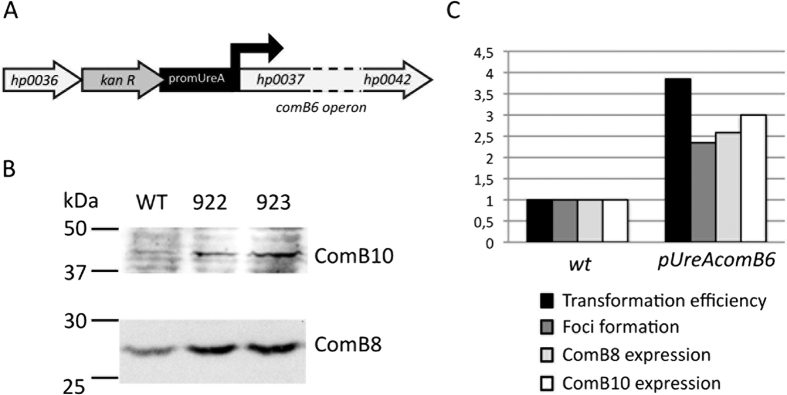
c*omB6* operon overexpression studies. (**A**) Schematic representation of the genomic construction for over expression of *comB6* operon. (**B**) Western blot against ComB10 or ComB8 of wild-type and two different strains constructed as indicated in A. (**C**) Correlation between transformation, foci formation and ComB8 expression in three independent clones overexpressing ComB8. In each experiment, all the values were reported to the value observed with the wt strain.

The results presented above suggested that the levels of ComB proteins, at least ComB8 and ComB10, could be a limiting factor for transformation in *H. pylori*. In order to test this hypothesis, we replaced in strain 26695 the promoter of the *comB6-B10* operon by the *ureA* strong promoter^38^ (Fig. 2A). We obtained 2 independent clones that present an average of 2.6-fold induction for ComB8 expression (Fig. 2B, and Table 3). Analysis of those clones revealed significant increases in the frequencies of transformation and of foci formation (Table 3 and Fig. 2C). These results show that the level of ComB complexes present in the bacterial membrane determines the amount of tDNA taken up by the bacteria and is a limiting factor for transformation.

### Transformation capacity, tDNA foci formation and ComB expression levels in different genetic backgrounds

**Figure 3:**
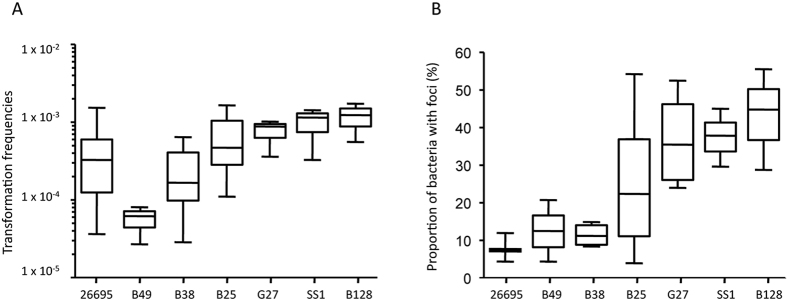
Transformation frequencies and foci formation in different strains. (**A**) Transformation frequencies. The bar represents the median, the boxes display the inner quartile range and the whiskers represent the minimum and maximum values. Number of experiments and p value determined with Mann-Whitney U test are indicated in Table 1. (**B**) Proportion of bacteria with foci. At least 3 independent experiments were analyzed and more than 1200 bacteria were counted for each strain. The percentage of bacteria harboring foci are indicated for the different strains. The bar represents the median, the boxes display the inner quartile range and the whiskers represent the minimum and maximum values.

Large variations in transformation frequencies have been reported amongst *H. pylori* isolates^39^. To determine if those differences are also a reflection of the DNA uptake capacities, we investigated the links between transformation, foci formation and ComB expression in different *H. pylori* genetic backgrounds. Because we monitored the replacement of a single nucleotide required for conferring streptomycin resistant, a major effect of restriction modification systems can be ruled out^40^. Moreover, the Mismach Repair pathway is absent in *H. pylori*^41^ and then cannot interfere in the experiment. The results presented in Table 4 and Fig. 3 show a general trend positively correlating transformation efficiencies with both, fraction of cells with foci (r^2^ = 0.75; p = 0.0115) and ComB8 expression (r^2^ = 0.59; p = 0.0426) (Fig. S1).

## Discussion

Several antibiotics induce genetic transformability in *S. pneumoniae*^28^. While some of them, like mitomycin C or fluoroquinones, induce DNA damage, others, like kanamycin and streptomycin, inhibit protein synthesis, suggesting that the induction of competence is a general stress response. In *H. pylori* there is contradictory data on the effect of antibiotics on competence. While an increase of transformation frequency was reported after ciprofloxacin exposure^27^, another group did not find an increase in the fraction of competent cells after treatment with either ciprofloxacin or mitomycin C^24^. Besides the fact that culture conditions used were different, such a discrepancy could also be explained by the fact that the end points determined were not the same, allowing the possibility that steps downstream of DNA uptake (i.e. DNA processing in the cytoplasm) are affected by the antibiotic treatments. Here, to avoid the pleiotropic effects of antibiotics, we used a genetic approach to analyse the impact of DNA damage on competence.

In a *H. pylori* strain deficient in AddA, a component of the nuclease/helicase recombination mediator AddAB, the expression of the genes coding for the competence T4SS is constitutively induced^27^. We had previously shown that inactivating genes coding for the components of the RecOR or AddAB mediator complexes, implicated in the formation of the RecA nucleofilament, leads to an increase in transformation frequencies^25^,^26^. Here, we extended these observations by showing that mutating *recN*, another gene involved in HR and DNA repair^33^,^42^, in strain 26695 also leads to increased transformation frequencies. As in most organisms, inactivation of HR in *H. pylori* sensitizes bacteria to agents inducing DNA strand breaks^25^,^26^,^33^,^43^,^44^,^45^. The increase in transformation frequencies in mutants affecting the initial steps of recombinational repair suggests that persistence of DNA strand breaks can be a cause for the enhancement of transformation capacity in *H. pylori.* Interestingly, disabling *uvrA* or *uvrB* genes, involved in the recognition of the lesion during nucleotide excision repair pathway, does not lead induction of transformation in *H. pylori*^46^. In *S. pneumoniae* it was suggested that a mutational burden itself could induce competence, possibly through the production of misfolded proteins^31^. Here, we show that in *H. pylori* the hypermutator phenotype obtained by inactivation of MutY, a protein implicated in base excision repair^35^, does not increase transformation frequencies (Table 1). Moreover, a *polA* mutant also deficient in DNA repair but displaying a hypomutator phenotype^34^ shows no modification of its transformation capacity. These results indicate that mutation frequency variations do not impact transformation capacity and suggest that it is the presence of persistent DNA breaks that is at the origin of the enhancement of transformability in *H. pylori.*

The lack of a known signalling pathway regulating competence in *H. pylori* raises the question of the mechanisms underlying the increased transformability observed in DNA repair deficient strains. Interestingly, the HR mutants *∆addA, ∆recO* and *∆recN*, that display higher transformability present higher levels of ComB8 and ComB10 proteins (Fig. 1), suggesting that the amount of the outer membrane ComB complex could be a limiting factor in the transformation process. This, together with the increased expression of *comB* genes transcripts in *addA* mutants^27^, indicates that the availability of the complex is likely to determine the DNA uptake capacity of the cell. Supporting this notion, overexpression of the *comB6-10* operon in strain 26695 leads to an increase in tDNA uptake, as reflected by foci formation, and in transformation frequencies (Fig. 2). Overexpression of ComB10 alone was not able to boost transformation^27^ indicating that at least another one of the proteins coded by the operon is required. In view of the results presented here, it is puzzling to note that *comB3* and *comB4* are themselves constitutively induced in *addA* strains^27^. A possible explanation for these somehow contradictory results is that there is a positive feedback mechanism regulating the expression of both operons as suggested by the enhanced expression of *comB9* in the *comB4* merodiploid^27^.

**Figure 4:**
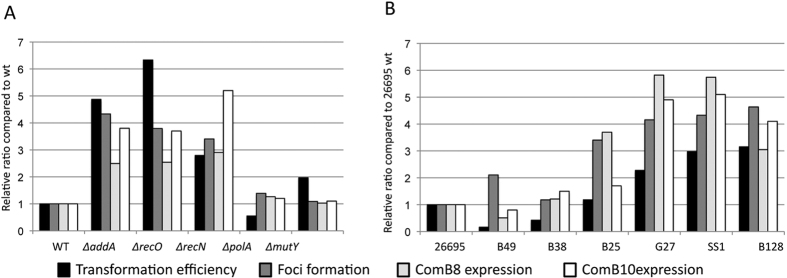
Comparison between transformation, foci formation, ComB8 and ComB10 expression in different strains. In each experiment, all the values were reported to the value observed with the wt 26695 strain. (**A**) Comparison in DNA repair mutants in 26695 background. (**B**) Comparison in different genetic backgrounds.

While the analysis of the relation between transformation frequencies, DNA foci formation and comB expression in the seven *wt* isolates shows a positive correlation between those parameters in most cases (Fig. 4 and Fig. 1S), in some strains other factors seem to be affecting the relative efficiencies of transformation. We cannot rule out differences in downstream steps of the transformation process such as the handling of the DNA once inside the cytoplasm. However the *comB6-10* operon overexpression experiment shows that all other factors being equal, the level of proteins coded by this operon is a limiting factor during transformation.

Taken together our results suggest a model by which the persistence of DNA breaks in the *H. pylori* genome induces the expression of the *comB6-10* operon, to increase the levels of the outer membrane ComB competence complex. That allows an enhanced uptake of exogenous DNA into the periplasm, which is a limiting step in the transformation process. In this model, however, the link between DNA damage and the induction of ComB expression remains to be determined.

## Methods

### Oligonucleotides, enzymes and reagents

Oligonucleotides used in this work were from Eurogentec. Restriction endonucleases, DNA polymerases, and DNA modifying enzymes were purchased from New England Biolabs. Culture media and antibiotics were from AES Chemunex and Sigma Aldrich, respectively. Fluorescent nucleotides Atto-448-dUTP (aminoallyl-dUTP) (Jena bioscience) were purchased from Euromedex and integrated in DNA by PCR as decribed before^11^.

### Strains and growth conditions

Except when indicated, all *Helicobacter pylori* strains were in 26695 background^47^ and are listed in Table S1. Plate cultures were grown at 37 °C in microaerophilic conditions (5% O_2_, 10% CO_2,_ using the MAC-MIC system from AES Chemunex) on blood agar base medium (BAB) supplemented with 10% defibrillated horse blood (AES) and an antibiotics mix. Plates were incubated from 24 h to 5 days depending on the experiment or the mutant selected. Liquid cultures were grown at 37 °C with gentle shaking under microaerophilic conditions in brain heart infusion media (BHI) supplemented with 10% defibrillated and de-complemented foetal bovine serum (Invitrogen).

To generate the corresponding mutant derivatives, the gene of interest (see list in Table S2) cloned with its flanking regions into pJET2.1 (Fermentas) was disrupted by a non-polar cassette carrying either kanamycin- (Km), apramycin- (Apra), or chloramphenicol- (Cm) resistance genes. DNA was introduced into *H. pylori* strains by natural transformation and mutants were selected by growth on either 20 μg ml^−1^ Km, 12,5 μg ml^−1^ Apra or 8 μg ml^−1^ Cm. Allelic replacement was verified by PCR.

### Construction of the strain overproducing *comB6-B10* operon

The construction of the integrative plasmid was based on single strand annealing technology as described elsewhere^48^ where the different elements have been amplified by PCR using overlapping primers. Primers listed in Table S3 displayed an overlap sequence ranging from 15 to 21 nucleotides with a Tm ranging from 49 °C to 63 °C.

Briefly, the 380 bp of the *hp0036* 3′ sequence was amplified with Hp0036_F primer that overlapped with pUC19_R and with Hp0036_R primer that overlapped with HpKanR_F primer. The non-polar cassette carrying either Km resistance gene was amplified with HpKanR_F and HpKanR_R overlapping with PromUreA_F. The *promUreA* domain corresponding to the 325 pb sequence directly upstream of *ureA* gene was amplified with PromUreA_F and PromUreA_R overlapping with HpComB6_F. The *hpComB6-1-133* domain was amplified with HpComB6_F and HpComB6_R overlapping with pUC19_F used to amplify pUC19. Finally, pUC19 was linearized with *Bam*HI and used as template for PCR amplification with pUC19_F and pUC19_R.All PCR were performed with Phusion DNA polymerase. After amplification, the PCR products were treated with *Dpn*I for parental template removal, and purified before assembly. Screening of correct plasmid was conducted by restriction and PCR and positive clones were confirmed by DNA sequencing.

The plasmid was introduced into *H. pylori* strains by natural transformation and mutants were selected by growth on 20 μg ml^−1^ Km, Allelic replacement was verified by PCR and sequenced. Overexpression of the operon was verified by Western Blot against ComB8 and ComB10.

### Natural transformation assay

200 ng of genomic DNA from strain LR133 (StrR)^25^ was mixed with 10^5^ cells from overnight plates resuspended in 15 μl of peptone water. Mixes were spotted on BAB plates. After 24 hours at 37 **°**C, dilutions of the resuspended spots were plated on BAB with and without the appropriate antibiotic (50 μg ml^−1^ Str) and incubated for 3 to 5 days. Transformation frequencies were calculated as the number of resistant colonies per recipient cfu.

### Western blot analysis of ComB8 and ComB10

Proteins from total cell extracts were separated by SDS-PAGE and transferred to nitrocellulose membranes. Membranes were incubated in blocking buffer (1X phosphate buffered saline [PBS] and 0.1% Tween 20 with 5% nonfat dry milk) overnight at 4 °C. Incubation with antibodies against either ComB8 or ComB10 (both provided by R. Haas, ref. 16) were carried out for 1 h at room temperature, followed by incubation with horseradish peroxidase (HRP)- conjugated secondary antibody under the same conditions. Proteins were revealed by adding ECL Prime Western blotting detection reagents (GE Healthcare) and read using the G-Box system (Syngene). Analysis of the correlation between ComB10 and ComB8 expression in all strains studied in this paper is significant (r2 = 0.6872 with a p value of 0,000459 (Fig. S1A).

### PCR DNA labelling

We used a 405 bp PCR fragment of the *rpsl* gene (hp1197) from 26696 mutated to give a Streptomycin resistance phenotype (LR133).

### Interaction of fluorescent DNA with *H. pylori*

To optimize images and to prevent cellular aggregation bacteria were recovered from exponential liquid cultures. 200 ng of ATTO labelled tDNA was mixed with 20 μl of cells (10^5^ cells) in BHI medium. The suspension was incubated for 10 min at 37 °C. The cells were then pelleted, washed once with 100 μl of BHI and resuspended in 20 μl of BHI.

### Microscopy assay on fixed bacteria

Samples from bacteria interacting with fluorescent ATTO-DNA were spotted on coated gelatin glass coverslips and fixed for 1 h with 4% formaldehyde. Slides were washed twice with PBS containing 0.1% glycine and once with PBS. Cell membranes were stained with FM64-X (Invitrogen) at a 1:500 dilution in PBS for 10 min and DNA was stained with 1 μg/ml 4,6-diamidino-2-phenylindole (DAPI). Coverslips were mounted in Dako fluorescent mounting medium. Image acquisition was performed with a Leica SPE confocal microscope (Wetzlar, Germany) using an ACS APO 63X (NA = 1.3) objective. Image treatment and analysis were performed using Leica and ImageJ software programs. All images presented correspond to a Z maximum intensity projection.

### Statistical analysis

Pairwise comparisons were performed using Mann-Whitney test, and correlations by Pearson test were measured on GraphPad Prism software.

## Additional Information

**How to cite this article:** Corbinais, C. *et al*. ComB proteins expression levels determine *Helicobacter pylori* competence capacity. *Sci. Rep.* **7**, 41495; doi: 10.1038/srep41495 (2017).

**Publisher's note:** Springer Nature remains neutral with regard to jurisdictional claims in published maps and institutional affiliations.

## Supplementary Material

Supplementary Tables

## Figures and Tables

**Table 1:** Transformation efficiency in DNA repair mutants.

strain genotype	n^a^	transformation frequency (×10^−4^)^b^ median (min; max)	Relative value	P value (MWU)
wt	31	3.9 (0.3; 26)	1	
*∆addA*	8	19.0 (5; 93)	4.9	0.0009
*∆recO*	11	24.7 (0.9; 114)	6.3	<0.0001
*∆recN*	7	10.9 (6; 31)	2.8	0.0003
*∆polA*	6	2.2 (0.3; 8)	0.6	0.1231
*∆mutY*	4	7.7 (0.3; 12)	2.0	0.3759
*∆comB6*	3	ND	0	

^a^Number of independent determinations (from 3 replicates mean in each experiment).

^b^Transformation tests were performed with genomic DNA conferring Streptomycin resistance (see Methods). The frequencies were calculated as the number of Str^R^ colonies per recipient CFU. Median are indicated; (min; max) correspond respectively to the minimum and the maximum values observed for each strain.

ND (Non Detected)MWU, Mann-Whitney U test between wt and the mutants.

**Table 2:** Foci formation, ComB8 and ComB10 expression in DNA repair mutants.

strain genotype	proportion (%)^a^ of bacteria with foci	foci formation (relative value)	P value^b^ (MWU)	ComB expression^c^ (relative value)	
	median (min; max)			ComB8	ComB10
wt	12.6 (4; 33)	1		1	1
*∆addA*	54.6 (46; 71)	4.3	<0.0001	2.5	3,8
*∆recO*	47.8 (31; 56)	3.8	<0.0001	2.5	3,7
*∆recN*	42.9 (33; 66)	3.4	<0.0001	2.9	5,2
*∆polA*	17.5 (14; 22)	1.4	0.2912	1.0	1,2
*∆mutY*	13.7 (9; 24)	1.1	0,5472	1.3	1,1
*∆comB6*	3.3 (0.9; 7)	0.3	0,0096	0	0

^a^At least 3 independent experiments were analyzed and more than 1200 bacteria were counted for each strain. Medians are indicated; (min; max) correspond respectively to the minimum and the maximum values observed for each strain.

^b^MWU, Mann-Whitney U test for foci formation between wt and the mutants.

^c^Medians of quantification from 5 to 15 Western blots from independent cultures are presented.

**Table 3:** Effect of *comB6-B10* operon overexpression on transformation frequency, foci formation and ComB8 expression.

strain	Transformation^a^		Foci formation^b^		ComB expression^c^ (relative value)	
	frequency (×10^−4^) median (min; max)	Relative value	Proportion, median (min; max)	Relative value	ComB8	ComB10
26695	3.9 (0.3; 26)	1	7.3 (4; 12)	1	1	1
pUreAComB6	15.0 (3; 45)	3,8****	17.1 (14; 23)	2.3***	2.6	3.0

^a^Transformation frequencies were calculated as the number of Str^R^ colonies per recipient CFU. ****p < 0.0001 with Mann-Whitney U test calculated between wt and the mutant.

^b^3 independent experiments were analyzed and more than 1200 bacteria were counted for each strain. ***p = 0.0002 with Mann-Whitney U test calculated between wt and the mutant.

^c^Medians of quantification from Western blots obtained with 3 independent strains are presented.

**Table 4:** Transformation frequency, foci formation, ComB8 and ComB10 expression in different strains.

strain	Transformation^a^		Foci formation^b^		ComB expression^c^ (relative value)	
	frequency (×10^−4^) median (min; max)	Relative value	Proportion, median (min; max)	Relative value	ComB8	ComB10
26695	3.9 (0.3; 26)	1	7.3 (4; 12)	1	1	1
B49	0.6 (0.3; 0.8)	0.2	15.4 (4; 21)	2.1	0.5	0.8
B38	1.7 (0.3; 6)	0.4	8.6 (4; 15)	1.2	1.2	1.5
B25	4.6 (1; 16)	1.2	24.8 (4; 54)	3.4	3.7	1.7
G27	8.9 (4; 10)	2.3	30.3 (23; 53)	4.2	5.8	4.9
SS1	11.6 (3; 14)	3.0	31.6 (26; 45)	4.3	5.7	5.1
B128	12.3 (6; 17)	3.2	33.9 (25; 55)	4.6	3.0	4.1

^a^Transformation frequencies were calculated as the number of StrR colonies per recipient CFU. Three independent experiments were analyzed for each strain.

^b^At least 3 independent experiments were analyzed and more than 1200 bacteria were counted for each strain.

^ab^Median are indicated; (min; max) correspond respectively to the minimum and the maximum values observed for each strain.

^c^Medians of quantification from 3 Western blots from independent cultures are presented.
