# Supplementary material for: ComB proteins expression levels determine Helicobacter pylori competence capacity
Source: Sci Rep. 2017 Jan 27;7:41495. doi: 10.1038/srep41495 (PMC5269756; doi:10.1038/srep41495)
Supplement: Supplementary Tables [file srep41495-s1.pdf]

Supplementary information

ComB proteins expression levels determine *Helicobacter pylori* competence capacity

Christopher Corbinais, Aurélie Mathieu, Prashant P. Damke, Thierry Kortulewski, Didier Busso, Mariano Prado-Acosta, J. Pablo Radicella and Stéphanie Marsin

Table S1

Table S2

Table S3

Supplementary references

Table S1: Correspondence between *H. pylori* open reading frame numbers and genes

gene	*comB6*	*addA*	*recO*	*recN*	*mutY*	*polA*
Orf number	Hp0037	Hp1553	Hp0951	Hp1393	Hp0142	Hp1470

Table S2: *H. pylori* Strains

Strain	Relevant Genotype	From
**LR1**	26695	[1]
**LR6**	SS1	[2]
**LR8**	B128	[3]
**LR10**	G27	[4]
**LR66**	B38	[5]
**LR67**	B25	[6]
**LR68**	B49	[6]
**LR133**	26695 str^R	[7]
**LR303**	26695 *addA::Cm*	[7]
**LR320**	26695 *addA::Apra*	[7]
**LR394**	26695 *recO::Km*	[7]
**LR320**	26695 *recO::Cm*	[7]
**LR118**	26695 *recN::Km*	This work
**LR317**	26695 *recN::Apra*	This work
**LR552/553**	26695 *mutY::Km*	This work
**LR366**	26695 *polA::Cm*	[8]
**LR531**	26695 *polA::Apra*	[8]
**LR922/923/925**	26695 *pUreA-comB6-Km*	This work
**LR711**	26695 *comB6::Km*	[9]

Table S3: Oligonucleotides used for the construction of *comB6* operon overproducing strains.

Name	*Sequence (5' -> 3')*
**Hp0036_F**	GCATGCCTGCAGGTCGACTCTAGAGCGATCAACCATCAAAAG
**Hp0036_R**	GTTAGTCACCCGGGTACCCAATACTTCAACGGACTTTAATG
**HpKanR_F**	GGTACCCGGGTGACTAAC
**HpKanR_R**	CTGAGCTACGCGACCGGATCCCCGGGTC
**PromUreA_F**	GGTCGCGTAGCTCAG
**PromUreA_R**	CTTATTCTCCTATTCTTAAAGTGTTTTTC
**HpComB6_F**	CTTTAAGAATAGGAGAATAAGATGAAAAATGACGCTTATG
**HpComB6_R**	GTTGTAAAACGACGGCCAGTGAATTCAAACTAAGGGCATAAAAACC
**pUC19_F**	GAATTCACTGGCCGTCG
**pUC19_R**	TCTAGAGTCGACCTGCAGG

Oligonucleotides are used as indicated in Methods. The annealing sequence is underlined.

1. Tomb, J. F. et al. The complete genome sequence of the gastric pathogen Helicobacter pylori. *Nature* **388**, 539-547 (1997).
2. Lee, A. et al. A standardized mouse model of Helicobacter pylori infection: introducing the Sydney strain. *Gastroenterology* **112**, 1386-1397 (1997).
3. Israel, D. A. et al. Helicobacter pylori strain-specific differences in genetic content, identified by microarray, influence host inflammatory responses. *J Clin Invest* **107**, 611-620 (2001).
4. Kimmel, B. et al. Identification of immunodominant antigens from Helicobacter pylori and evaluation of their reactivities with sera from patients with different gastroduodenal pathologies. *Infect Immun* **68**, 915-920 (2000).

5. Thiberge, J. M. et al. From array-based hybridization of Helicobacter pylori isolates to the complete genome sequence of an isolate associated with MALT lymphoma. *BMC Genomics* **11**, 368 (2010).
6. Bussiere, F. I. et al. H. pylori-induced promoter hypermethylation downregulates USF1 and USF2 transcription factor gene expression. *Cell Microbiol* **12**, 1124-1133 (2010).
7. Marsin, S., Mathieu, A., Kortulewski, T., Guerois, R. & Radicella, J. P. Unveiling novel RecO distant orthologues involved in homologous recombination. *PLoS Genet* **4**, e1000146 (2008).
8. Garcia-Ortiz, M. V. et al. Unexpected role for Helicobacter pylori DNA polymerase I as a source of genetic variability. *PLoS Genet* **7**, e1002152 (2011).
9. Corbinais, C., Mathieu, A., Kortulewski, T., Radicella, J. P. & Marsin, S. Following transforming DNA in Helicobacter pylori from uptake to expression. *Mol Microbiol* **101**, 1039-1053 (2016).
